# The effect of nasal douching by hypertonic 2.3 per cent sea water with algae extracts on the concentration of epidermal growth factor, transforming growth factor-α and interleukin-8 in nasal secretions of patients with nasal polyposis following endoscopic surgical treatment

**DOI:** 10.1017/S0022215123001974

**Published:** 2024-05

**Authors:** Aleksandar Perić, Dejan Gaćeša, Sandra Vezmar Kovačević, Aneta V. Perić, Danilo Vojvodić, Stella Georgiou, Evdokia Protopapadakis, Konstantinos Alevizopoulos

**Affiliations:** 1 Department of Otorhinolaryngology, Faculty of Medicine of the Military Medical Academy, University of Defense, Belgrade, Serbia; 2 ENT Hospital “Dr Žutić”, Belgrade, Serbia; 3 Department of Pharmacokinetics and Clinical Pharmacy, Faculty of Pharmacy, University in Belgrade, Belgrade, Serbia; 4 Institute for Pharmacy, Faculty of Medicine of the Military Medical Academy, University of Defense, Belgrade, Serbia; 5 Institute for Medical Research, Division of Clinical and Experimental Immunology, University of Defense, Belgrade, Serbia; 6 Research and Development Department, Gerolymatos International S.A., Athens, Greece

**Keywords:** Cytokines, nasal lavage fluid, nasal mucosa, nasal polyps, nasal surgical procedures, saline solution

## Abstract

**Objective:**

To investigate epidermal growth factor, transforming growth factor-α and interleukin-8 production in nasal mucosa irrigated with hypertonic 2.3 per cent solution with algae extracts, in comparison to 0.9 per cent NaCl during the first two weeks after surgery for nasal polyposis, in relation to symptoms and local findings.

**Methods:**

This prospective study included 20 nasal polyposis patients postoperatively irrigated with hypertonic solution and 20 nasal polyposis patients postoperatively irrigated with isotonic solution. We evaluated nasal symptom score, endoscopic score and mediator levels in nasal secretions before and after irrigation.

**Results:**

Following treatment, nasal symptom score and endoscopic score were significantly lower in the hypertonic solution group (*p* = 0.023; *p* < 0.001, respectively). The increase in the epidermal growth factor and the decrease in the transforming growth factor-α and interleukin-8 concentration were higher in the hypertonic group (*p* < 0.001 for all mediators).

**Conclusion:**

Irrigation with a hypertonic solution was found to be more effective than an isotonic solution in nasal mucosa reparation.

## Introduction

Nasal polyposis or chronic rhinosinusitis with nasal polyps is a chronic inflammation of the mucous membrane of the nasal cavity and paranasal sinuses, characterised by T2 immune response and eosinophil infiltration of the epithelium and oedematous stroma in more than 90 per cent of patients in the European, North American and Australian populations.^1,2^ This disease, despite numerous studies, still does not have a clear answer in terms of aetiology and pathogenesis. Staphylococcal enterotoxins, bacterial biofilm, allergy, arachidonic acid metabolism disorder, impaired immune barrier, and others are still under consideration.^1,2^ According to the results of numerous studies, functional endoscopic sinus surgery (FESS), administration of topical and systemic corticosteroids, long-term low-dose use of macrolide antibiotics and doxycycline, as well as biologic therapy are the principles of nasal polyposis treatment.^1–5^ Also, according to modern guidelines for the treatment of chronic rhinosinusitis, rinsing the nasal cavity with saline solutions is a mandatory part of the therapy, especially in the period after FESS.^1,2,6^

Although the efficacy of FESS has been confirmed within the past three decades, delayed nasal mucosal healing and formation of adhesions following FESS, especially in the region of the middle nasal meatus, can be a potential cause of surgical failure, including blockage of mucociliary clearance and the recurrence of the sinus disease.^7^ In the period after FESS, large areas of de-epithelialised nasal and/or paranasal sinus mucosa are left behind, and therefore it is necessary to ensure better epithelialisation of the mucosa and prevent the formation of granulations, fibrosis, crusts and adhesions in that sensitive period of two to three weeks. One of the key moments in that process is to provide moisture to the mucosa, which is achieved by douching with saline solutions, which can be divided into isotonic and hypertonic. Both have been shown to be effective during previous research.^1,2^ Hypertonic 2.3 per cent NaCl solutions combine two primary actions: (1) mechanical cleansing and removal of mucus and harmful agents resulting in flushing out dust, debris, pollutants, allergens, germs, inflammatory mediators and excess mucus from the nose and sinuses; and (2) osmotic action due to the difference in ionic (salt) content between the sprayed liquid and the congested nasal tissues that will cause natural decongesting effects.^8^ In patients with nasal polyposis associated with aspirin-exacerbated respiratory disease, nasal douching by hypertonic (2.3 per cent) sea water after FESS was found to be superior compared to isotonic (0.9 per cent) saline.^9^ Patients using 2.3 per cent sea water felt less nasal obstruction, facial pain and/or pressure, headache and trouble sleeping. In the endoscopic findings from the nasal cavity, patients showed less nasal mucosal oedema, nasal secretion and crusting comparing to aspirin-exacerbated respiratory disease patients using isotonic saline.^9^ Patients after septoplasty and radiofrequency turbinate volume reduction reported less nasal bleeding and less crust formation if they used hypertonic 2.3 per cent solution enriched with extracts of two algae (brown alga *Undaria pinnatifida* and blue-green alga *Spirulina platensis*) than when they used 0.9 per cent saline.^10^

Previous studies were concerned mainly with the evaluation of symptoms and local findings during the assessment of the effectiveness of different forms of saline nasal irrigation. Investigation of biochemical parameters in nasal secretions was mostly not in focus. Given that the biochemical composition of nasal secretions quite faithfully reflects the inflammatory status of the nasal mucosa, examination of these parameters could show the state of the mucosal membrane during the healing process of mucosal wounds after FESS for nasal polyposis.^11^ As a key mediator in wound healing, epidermal growth factor is an effective mitogenic agent in epithelial cells, fibroblasts and vascular endothelial cells. According to previous investigations, epidermal growth factor significantly enhances the proliferation and migration of ciliated respiratory cells as well as increases the ciliary beating frequency.^12^ Transforming growth factor-α has diverse pathophysiologic functions, including immunological, inflammatory and neoplastic processes. It is closely related to epidermal growth factor and they both act on the epidermal growth factor-receptor to promote the differentiation and proliferation of epithelial cells, angiogenesis, fibroblast proliferation and wound healing.^13^ Besides epithelial cells and fibroblasts, eosinophils and macrophages have also been found to express transforming growth factor-α.^13^ Lam *et al*. found that transforming growth factor-α showed similar immunohistochemical localisation in both allergic rhinitis, perennial and seasonal.^13^ Interleukin (IL)-8, also known as neutrophil activating peptide, CXCL8, and granulocyte chemotactic protein-1, is a cytokine that belongs to the CXC chemokine family. It plays a pivotal role in the pathophysiology of inflammation during the infection.^14^ IL-8 is a powerful chemoattractant for neutrophils, basophils and T cells. As a regulator of inflammation related to bacterial infection, it has an important role in the process of wound healing during the nasal mucosa reparative process.^14^

The aim of this study was to investigate the production of epidermal growth factor, transforming growth factor-α and IL-8 in nasal mucosa irrigated with hypertonic 2.3 per cent sea water with the addition of algae (*Undaria pinnatifida* and *Spirulina platensis*) extracts, in comparison to isotonic 0.9 per cent sodium chloride solution in the reparative phase during the first two weeks after FESS for nasal polyposis, in relation to symptoms and local findings in the nasal cavity.

## Materials and methods

### Study design

This study was designed as a prospective, single centre, real-life, open-label, non-randomised and non-interventional study. The study was conducted between September 2022 and June 2023 in our Department of Otorhinolaryngology, according to the principles published in the Helsinki Declaration and with Institutional Review Board Approval (Military Medical Academy Ethics Committee Approval N^o^ 21/2022). It was performed as a part of the scientific project (MFVMA02/23-25/) of our institution. Written informed consent was obtained by all participants.

### Study participants

The study included patients diagnosed with chronic rhinosinusitis with nasal polyps, according to the European Position Paper on Rhinosinusitis and Nasal Polyps (EPOS) 2020 guidelines,^1^ whose Lund–Mackay computed tomography (CT) score^15^ was 15 and above. All patients underwent bilateral fronto-spheno-ethmoidectomy for nasal polyposis with the same extent of surgery. In patients with significant deformation of the nasal septum, septoplasty was performed, and in those with pneumatisation and enlargement of the middle turbinate (concha bullosa), lateral resection of the concha bullosa was performed. All operations were made by the same surgeon using the same surgical technique. Patients with asthma and hypersensitivity to non-steroidal anti-inflammatory drugs were not included due to the possible need to take topical and/or systemic corticosteroid therapy immediately before and after surgical treatment.

The other exclusion criteria were (1) patients younger than 18 and older than 65 years of age, (2) patients with antrochoanal polyps, hamartomas, systemic diseases that manifest in the nose and sinus area; (3) pregnant and/or breastfeeding patients; (4) patients with acute inflammation of the upper and lower airway; and (5) use of topical and systemic corticosteroids, antibiotics and antihistamines by patients within four weeks before the study.

### Preoperative CT and symptom assessment

Preoperatively, the degree of disease extension on CT scans of the paranasal sinuses was assessed based on the Lund–Mackay CT score^15^ a day before surgery. Then, a preoperative assessment of the intensity of nasal symptoms was performed by the patients based on a visual analogue scale (0–10 cm; 0 cm = absent to 10 cm = maximum intensity) of 5 symptoms: nasal obstruction, nasal secretion and/or postnasal discharge, sense of pressure in the face, headache and loss of the sense of smell. The nasal symptom score was calculated as the sum of these symptoms. Antibiotic prophylaxis consisted of a single dose of cefuroxime 1.5 g, two hours before the start of surgery.

### Postoperative care

After FESS, nasal packing (cotton wool gauze packs with Vaseline ointment) was put bilaterally in the nasal cavity. On the third day, the nasal packing was removed, and the patients were instructed how to start nasal irrigation. According to the principle of established motivation for using hypertonic or isotonic solutions, the patients were successively divided into two groups of 20 subjects each. All patients were familiar with the beneficial effects and potential side effects of each of the preparation and were familiar with which preparation they were using. Group 1 used hypertonic seawater (2.3 per cent NaCl, composition NaCl 23.00 g/l, sulphates 2.30 g/l, magnesium 1.04 g/l, calcium 0.31 g/l, potassium 0.42 g/l, copper traces, zinc traces) with extracts of brown algae (*Undaria pinnatifida*) and blue-green algae (*Spirulina platensis*) (Sinomarin® Plus Algae ENT, Gerolymatos International S.A., Athens, Greece). Group 2 used isotonic sodium chloride (0.9 per cent NaCl) (Esensa d.o.o., Belgrade, Serbia). All subjects were asked and instructed to use nasal douching solutions three times daily for a period of 14 days following removal of the nasal packing.

### Clinical evaluation after FESS

Patients were evaluated by the physician on the 1^st^ and 17^th^ days after the removal of the nasal packing. Patients recorded their symptoms in diary cards twice a day, in the morning and in the evening, and nasal symptom score was assessed on the 1st day and the 17th day. At each visit, the same physician used a 4 mm, 0° and 30° endoscope (Karl Storz – Endoscope SE & Co, Tuttlingen, Germany) to evaluate the intensity of mucosal oedema, nasal secretion and nasal crusting, and rated their local findings as follows: 0, none; 1, mild; 2, moderate; 3, moderately severe; and 4, severe. The endoscopic score was calculated as the sum of these three endoscopic signs.

### Nasal secretion sampling and immunochemistry

Nasal secretion samples were taken in all 40 patients, the day before FESS, on the 1st day and on the 17th day after removal of the nasal packing (i.e. on the 3rd day after the end of the nasal douching) in order to allow the nasal mucosa in the regeneration phase to excrete a sufficient amount of inflammatory mediators unhindered. The absorption technique with cotton wool sticks (length 10 mm, width 4 mm) was used for sampling, as previously described.^16^ Sticks were placed in the middle nasal meatus for 5 minutes, which is as long as it takes for the cotton wool to be completely soaked with nasal secretions. Then, the cotton wool with the samples was placed in Eppendorf tubes containing 1 ml of transfer medium for 30 minutes, which is as long as it takes for the mediators to diffuse into the solution of transfer medium. After centrifugation, the supernatants were frozen at −70°C and stored until the detection of mediators. We measured the concentrations (expressed in picograms per millilitre (pg/ml)) of epidermal growth factor, transforming growth factor-α and IL-8 in each of the samples, using commercial human enzyme-linked immunosorbent assay kits (R & D Systems, Inc, Minneapolis, USA). The detection ranges for mediators are as followed: 3.91–250 pg/ml for epidermal growth factor, 1.37–1000 pg/ml for transforming growth factor-α and 15.6–1000 pg/ml for IL-8.

### Potential adverse effects

The investigator queried all participants for any particular adverse events (nasal irritation, burning sensation, facial pain), with severity grades as mild, moderate and severe.

### Statistical analysis

The parameters were expressed as mean ± standard deviation. For between-group comparison, we used the non-parametric Mann–Whitney U test. For paired comparisons within a group, we used the Wilcoxon signed rank test. The statistical significance (*p*) was set at the level of 0.05. The analysis was performed using version 19.0 of the Statistical Package for the Social Sciences (SPSS) software (SPSS Inc., Chicago, USA).

## Results and analysis

Forty patients with nasal polyposis were included in the study. Of these, 20 patients rinsed their nasal cavities after FESS with a 2.3 per cent hypertonic solution with algae extracts, and 20 patients rinsed with an isotonic 0.9 per cent sodium chloride solution. There was no statistically significant difference between the two groups in the demographic and baseline clinical and biochemical parameters (Table 1). After douching with saline solutions, nasal symptom score and endoscopic score were significantly lower in the group that used the hypertonic solution compared with the group that used the isotonic solution (*p* = 0.023; *p* < 0.001, respectively) (Table 2).

**Table 1.** Comparison of demographic and baseline clinical and biochemical parameters between hypertonic 2.3 per cent with algae extracts solution and sodium chloride 0.9 per cent group

Parameter	Hypertonic 2.3% with algae extracts solution	Sodium chloride 0.9% solution	*p*-value
Age	46.70 ± 9.48	44.60 ± 8.07	0.386
Gender (% female)	50	50	1.000
NSS	39.90 ± 3.06	40.60 ± 2.84	0.683
LMS	18.35 ± 2.21	19.35 ± 1.63	0,174
EGF (pg/ml)	123.53 ± 33.53	130.13 ± 30.73	0,507
TGFα (pg/ml)	622.80 ± 129.86	557.60 ± 80.89	0.114
IL-8 (pg/ml)	415.77 ± 150.69	357.60 ± 118.06	0.185

Abbreviations: EGF, epidermal growth factor; TFG-α, transforming growth factor alpha; IL-8, interleukin-8; NSS, nasal symptom score; LMS, Lund-Mackay computed tomography (CT) score. All results are presented as mean ± standard deviation.

**Table 2.** Comparison of clinical and biochemical parameters between hypertonic 2.3 per cent with algae extracts solution and sodium chloride 0.9 per cent solution group after FESS

Parameter	Hypertonic 2.3% with algae extracts solution	Sodium chloride 0.9% solution	*p*-value
NSS Post	16.80 ± 3.32	14.90 ± 2.63	0.037
NSS Final	7.90 ± 1.52	8.45 ± 1.64	0.023
ES Post	5.85 ± 0.67	5.95 ± 0.67	0.641
ES Final	3.05 ± 0.60	4.00 ± 0.65	<0.001
EGF Post (pg/ml)	37.24 ± 4.19	55.64 ± 22.69	0.006
EGF Final (pg/ml)	115.19 ± 36.32	61.03 ± 19.90	<0.001
TGFα Post (pg/ml)	137.73 ± 41.23	117.63 ± 33.00	0.176
TGFα Final (pg/ml)	55.34 ± 16.52	84.37 ± 21.59	<0.001
IL-8 Post (pg/ml)	364.69 ± 89.81	332.43 ± 137.87	0.273
IL-8 Final (pg/ml)	65.01 ± 21.74	116.59 ± 43.92	<0.001

Abbreviations: EGF, epidermal growth factor; TFG-α, transforming growth factor alpha; IL-8, interleukin-8; NSS, nasal symptom score; ES, endoscopic score; Post, after the removal of the nasal packs (after surgery); Final, three days after the end of the nasal douching. All results are presented as mean ± standard deviation.

**Figure 1.**
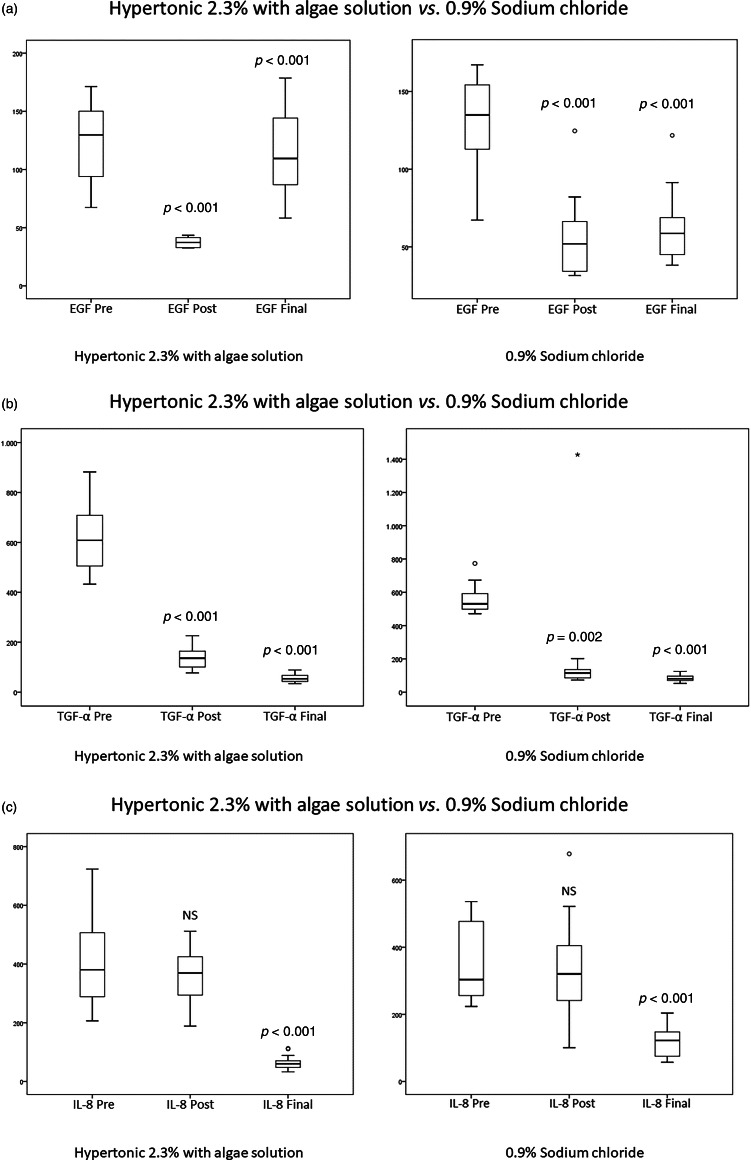
Concentrations of (a) EGF, (b) TGF-α and (c) IL-8 in nasal secretion samples in all three measurement points. Abbreviations: EGF, epidermal growth factor; TGF-α, transforming growth factor alpha; IL-8, interleukin 8; Pre, before surgical treatment; Post, after the removal of nasal packing; Final, three days after the end of nasal douching. All concentrations of biochemical parameters are expressed in pg/ml.

**Figure 2.**
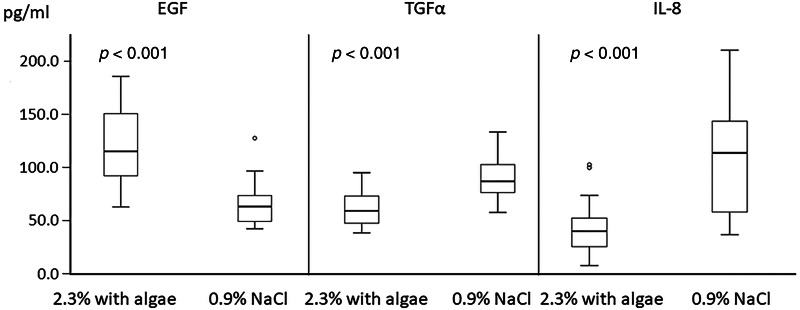
Concentrations of EGF, TGF-α and IL-8 in nasal secretion samples on 17th day after the removal of nasal packing (3rd day after the end of nasal douching). EGF level is significantly higher and TGF-α and IL-8 are significantly lower in the hypertonic 2.3 per cent with algae extracts solution group than in the sodium chloride 0.9 per cent solution group (*p* < 0.001 for all parameters). Abbreviations: EGF, epidermal growth factor; TGF-α, transforming growth factor alpha; IL-8, interleukin 8.

Regarding biochemical parameters, after removal of the nasal packing, concentrations of epidermal growth factor and transforming growth factor-α were significantly lower in comparison to levels before FESS (*p* < 0.001; *p* < 0.001, respectively for the hypertonic solution group) (*p* < 0.001; *p* = 0.002, respectively for the isotonic solution group) (Figure 1a, b). We found no significant difference between concentrations of IL-8 in nasal secretion before FESS and after the nasal packing removal (Figure 1c). Three days after the end of nasal douching, epidermal growth factor was significantly higher, while transforming growth factor-α and IL-8 were significantly lower in the nasal secretions of both hypertonic and isotonic solution groups (*p* < 0.001 for all three parameters) (Figure 1a–c). However, the increase in concentration of epidermal growth factor as well as the decrease in concentration of transforming growth factor-α and IL-8 after the end of the nasal douching were significantly higher in the group of patients who rinsed the nasal cavities with a hypertonic solution with algae extracts (*p* < 0.001 for all three parameters) (Table 2, Figure 2). No side effects were recorded in any of the studied groups.

## Discussion

Based on results of a previous prospective study, nasal douching with 2.3 per cent sea water, supplemented with seaweed extracts (*Undaria pinnatifida* and *Spirulina platensis*) and dex-panthenol was found to be effective as an add-on therapy for children and adolescents with allergic rhinitis.^17^ Another study showed that nasal irrigation with 2.3 per cent solution, enriched with brown algae and blue-green algae extracts as well as essential oils of *Eucalyptus globulus*, *Mentha spicata* and *Thymus vulgaris* extracts is a safe and effective method for reducing symptoms of acute viral rhinosinusitis, caused by severe acute respiratory syndrome coronavirus-2 (SARS-CoV-2).^18,19^ This confirmed a previous strategy of successfully using nasal douching solutions in reduction of symptoms of acute viral infection of the upper airway. During irrigation of the nasal cavity with saline solutions, viral particles are mechanically removed. Under the influence of Cl^−^ ions in a hypertonic solution, the conformation of the angiotensin-converting enzyme 2 receptor occurs, which hinders binding of the SARS-CoV-2 to the nasal epithelium of the host.^20^ An increased concentration of NaCl leads to the activation of the enzyme furin protease, which breaks down the ‘spike protein’ of the virus.^20^ Sulphurised fucoidan, isolated from the alga *Undaria pinnatifida* in *in vitro* conditions interferes with the binding process of SARS-CoV-2 to host cells and inhibits cellular inflammatory infiltrate.^21^ *Spirulina platensis* extract blocks the penetration of SARS-CoV-2 into the host cell by an unknown process.^21^

Our results suggest that the concentrations of epidermal growth factor and transforming growth factor-α in nasal secretions decrease after FESS in nasal polyposis patients. Epidermal growth factor is the main growth factor that stimulates epithelial proliferation and is highly presented in the pathogenesis of nasal polyposis.^7,12^ Transforming growth factor-α has a role that is complementary to its similar mediator, transforming growth factor-β. Both mediators trigger the remodelling process of the nasal and paranasal sinuses mucosa in patients with chronic rhinitis and chronic rhinosinusitis, which can lead to the formation of nasal polyposis.^13^

Transforming growth factor-β, which has been recognised as a chemoattractant for fibroblasts and a promoter of fibroblast proliferation, is present only in inflammatory polyps, but absent in normal nasal mucosa.^13^ Like transforming growth factor-β, transforming growth factor-α has been thought to stimulate fibroblasts and epithelial cell proliferation. In fact, transforming growth factor-β must be present along with transforming growth factor-α to stimulate proliferation of fibroblasts.^13^ Homma *et al*.^22^ found that exposure or colonisation by *Staphylococcus aureus* in the airway may enhance the remodelling of tissue through a transforming growth factor-α-dependent induction of matrix metalloproteinase-1 expression and may, thereby, promote remodelling in airway diseases in which *Staphylococcus aureus* is implicated, such as asthma and chronic rhinosinusitis with nasal polyps. Also, transforming growth factor-α is highly expressed in nasal mucosa of chronic rhinosinusitis with nasal polyps and is deeply involved in MUC5AC gene induction and in mucus hypersecretion.^23^ During endoscopic polypectomy, the epithelium, eosinophils, fibroblasts, vascular endothelium and macrophages, as rich sources of epidermal growth factor and transforming growth factor-α, are removed, which leads to a decrease in the concentration of these mediators in nasal secretions after removal of the nasal packing.

IL-8 is one of the mediators that regulates inflammation during bacterial infection.^14^ According to the results of our study, its concentration in the nasal secretions did not change after removing the nasal packing. Large wound surfaces on the nasal mucosa after FESS and blockage of mucociliary transport by nasal packing can lead to the potential development of bacterial infection, which favour the production of IL-8. This could explain the fact that IL-8 concentration does not decrease in nasal secretions after FESS.

Our results suggest that rinsing the nasal cavity by hypertonic sea water with the addition of algae extracts leads to a better improvement of symptoms and endoscopic findings, including less swelling of the nasal mucosa, less amount of thick mucus and less-pronounced crust formation on the mucosa. Hypertonic sea water also leads to a significantly greater increase in epidermal growth factor concentration and a significantly greater decrease in transforming growth factor-α and IL-8 compared to isotonic solution.

Wounded nasal mucosa is susceptible to bacterial infection and the wound healing process is a type of acute inflammation, which results in the environment of the nasal secretions becoming alkaline.^8^ By using hypertonic 2.3 per cent solution, acidification of the environment occurs, which makes reproduction of microorganisms difficult. As a result, fibroblasts and macrophages secrete a smaller amount of IL-8, a chemokine which serves as an attractant for neutrophils,^8^ leading to a lower concentration of IL-8 in the mucus on the 17th day after the removal of the nasal packing. The absence of infection also leads to an undisturbed epithelialisation process.^7,12^ Epithelial cells secrete new amounts of epidermal growth factor, which results in a higher level of epidermal growth factor in the mucus after douching with a hypertonic solution.

It is very important to take account of the significance of algae extracts in the wound-healing process after FESS. An experimental study by Chen *et al*.^24^ showed that fucoidan from *Undaria pinnatifida* could promote the repair of epidermal barrier disruption in mice. The blue-green microalgae *Spirulina platensis* gained more attention for its antioxidant and anti-inflammatory properties and roles in the process of wound healing. A study by Ebrahimi *et al*.^25^ examined the effects of *Spirulina platensis* on cutaneous wound repair in mice. Histopathological examination revealed higher level of inflammatory cell infiltration, fibroblast proliferation, angiogenesis, epithelialisation, extracellular matrix deposition and better wound contraction in mice treated with *Spirulina platensis* as compared to a control group.^25^ In this way, wound healing is accelerated through the improvement of angiogenesis and collagen production.^25^ Therefore, we speculate that addition of these two algae extracts to the hypertonic 2.3 per cent solution possibly could be very useful as a biomedical application to treat various wounds of the nasal mucosa after FESS.

By reviewing the literature, we found one article that dealt with the concentrations of growth factors in nasal secretions after FESS. Results of study, which was conducted by Watelet *et al*.^26^, showed a tendency to increase the concentrations of transforming growth factor-β1 and transforming growth factor-β2 and decrease the concentration of epidermal growth factor after nasal douching following FESS, which is contrary to our results. However, a careful analysis of the study reveals numerous methodological differences compared to our current one. The groups in the previous study are not homogeneous; apart from patients with nasal polyposis, there are also those without nasal polyposis. After FESS, the nasal cavities were rinsed with an isotonic solution, without the addition of medicinal algae.^26^ All patients started with intranasal corticosteroid spray fluticasone propionate immediately after FESS.^26^ At our institution, there is a protocol to administer intranasal corticosteroid sprays in parallel with saline irrigation immediately after nasal packing removal only in patients with nasal polyposis associated with aspirin-exacerbated respiratory disease, as shown in a previous study.^9^ The more severe clinical course of the disease and the high association with relapses are the main raisons for protocol.^9^ However, patients with this clinical entity within chronic rhinosinusitis were not included in our current study. It is possible that simultaneous application of corticosteroids and isotonic solution affects the healing process of the nasal mucosa differently than when only rinsing with a hypertonic solution with the addition of algae extracts is applied. In addition, in the study of Watelet *et al*.^26^, after finishing nasal irrigation after three weeks, a few days were not left for the nasal mucosa to excrete a sufficient level of mediators.

- The biochemical composition of nasal secretions faithfully reflects the inflammatory status of the nasal mucosa
- Previous studies were mainly concerned with the evaluation of symptoms and local findings during the assessment of the effectiveness of different forms of saline nasal irrigation
- The examination of inflammatory mediators in nasal fluid could show the state of the nasal mucosa during the healing process of mucosal wounds after endoscopic sinus surgery for nasal polyposis
- The results of this study suggest that postoperative douching the nasal cavity with a hypertonic 2.3% solution with the addition of medicinal algae *Undaria pinnatifida* and *Spirulina platensis* is more effective than isotonic solution in improving symptoms and local findings
- A significantly higher increase in the concentration of epidermal growth factor and a decrease in the levels of transforming growth factor-α and interleukin-8 in the mucus indicate a better effects of hypertonic sea water in the direction of healing of the nasal mucosa after surgical treatment

Our study has some limitations, given that it was designed as non-randomised, and it evaluated the effects on a relatively small number of subjects. Our study was not a double-blind and placebo-controlled study.

## Conclusion

The results of this study suggest that douching the nasal cavity with a hypertonic solution with the addition of medicinal algae after FESS in patients with nasal polyposis is more effective than isotonic solution in improving symptoms and local findings. A significantly higher increase in the concentration of epidermal growth factor and a decrease in the levels of transforming growth factor-α and IL-8 in the mucus indicate better effects of hypertonic sea water toward healing of the nasal mucosa after FESS.
